# A holistic framework integrating plant-microbe-mineral regulation of soil bioavailable nitrogen

**DOI:** 10.1007/s10533-021-00793-9

**Published:** 2021-05-06

**Authors:** Amanda B. Daly, Andrea Jilling, Timothy M. Bowles, Robert W. Buchkowski, Serita D. Frey, Cynthia M. Kallenbach, Marco Keiluweit, Maria Mooshammer, Joshua P. Schimel, A. Stuart Grandy

**Affiliations:** 1 Department of Natural Resources and the Environment, University of New Hampshire, 56 College Road, Durham, NH 03824 USA; 2 Department of Plant and Soil Sciences, Oklahoma State University, Stillwater, OK USA; 3 Department of Environmental Science, Policy, and Management, University of California Berkeley, Berkeley, CA USA; 4 Department of Biology, University of Western Ontario, London, ON Canada; 5 Department of Natural Resource Sciences, McGill University, Montreal, Canada; 6 School of Earth & Sustainability and Stockbridge School of Agriculture, University of Massachusetts, Amherst, MA USA; 7 Department of Ecology, Evolution, and Marine Biology, University of California, Santa Barbara, CA USA

**Keywords:** Depolymerization, Particulate organic matter, Mineral associated organic matter, Microbial physiology, Fertilizer

## Abstract

Soil organic nitrogen (N) is a critical resource for plants and microbes, but the processes that govern its cycle are not well-described. To promote a holistic understanding of soil N dynamics, we need an integrated model that links soil organic matter (SOM) cycling to bioavailable N in both unmanaged and managed landscapes, including agroecosystems. We present a framework that unifies recent conceptual advances in our understanding of three critical steps in bioavailable N cycling: organic N (ON) depolymerization and solubilization; bioavailable N sorption and desorption on mineral surfaces; and microbial ON turnover including assimilation, mineralization, and the recycling of microbial products. Consideration of the balance between these processes provides insight into the sources, sinks, and flux rates of bioavailable N. By accounting for interactions among the biological, physical, and chemical controls over ON and its availability to plants and microbes, our conceptual model unifies complex mechanisms of ON transformation in a concrete conceptual framework that is amenable to experimental testing and translates into ideas for new management practices. This framework will allow researchers and practitioners to use common measurements of particulate organic matter (POM) and mineral-associated organic matter (MAOM) to design strategic organic N-cycle interventions that optimize ecosystem productivity and minimize environmental N loss.

**Supplementary Information:**

The online version contains supplementary material available at 10.1007/s10533-021-00793-9.

## Introduction

Nitrogen (N) is essential for life as a key constituent of biomolecules including DNA, RNA, chlorophyll, and enzymes. In soil, bioavailable N is comprised of dissolved inorganic and organic N—including small polymers and monomers—that can be assimilated by plants and/or microbes. Supplies of bioavailable soil N sometimes exceed plant requirements, but often fail to meet them, resulting in N asynchrony that constrains ecosystem productivity and exacerbates environmental nutrient losses, which are expected to intensify under climate change (Sinha et al. 2017; Bowles et al. 2018; Houlton et al. 2019; Dai et al. 2020). This “N problem” arises in part because of nitrogen's changeable nature: as a reactive element found in multiple forms and seven oxidation states, N is difficult to track and manage.

Unresolved issues in intensively managed agroecosystems epitomize our incomplete understanding of bioavailable N. In these systems, the persistent challenge of minimizing N losses and improving the spatial and temporal match between N availability and plant N demand (i.e. N synchrony) derives in part from a historical focus on the inorganic N pool. Even with high synthetic N inputs, however, a substantial fraction of inorganic N is derived from the soil organic matter pool (Yan et al. 2020). Yet, we remain without a universal and accurate assay or model that can predict organic N (ON) conversion to plant-available inorganic N, despite the long-acknowledged need for one (e.g. Vitousek 1982; Schimel and Bennett 2004) and continuing efforts to develop a suitable N availability index (Ros 2012; Curtin et al. 2017; Clivot et al. 2017; McDaniel et al. 2020).

A focus on inorganic N pools overlooks the important mechanisms occurring in soil that determine how much ON feeds into and supplies the inorganic N pool. Moreover, the ON component of the bioavailable N pool is itself a critical N source to plants and microbes. Estimates of bioavailable N that do include ON usually represent it as the short-term potentially mineralizable N pool. However, this pool is operationally defined; in measuring net changes in inorganic N under optimized conditions and in the absence of live plant roots, potentially mineralizable N often poorly explains the variability in outcomes such as crop yields, estimated or actual crop N availability, and fertilizer needs (Fox and Piekielek 1984; Thicke et al. 1993; Curtin and McCallum 2004; Dessureault-Rompré et al. 2014; McDaniel et al. 2020). Agricultural practitioners currently rely on N-credit calculators that do not explicitly consider soil processes and interactions (Lory et al. 1995) and are prone to uncertainty, bias, and error (Sharma and Bali 2018). The struggle to quantify the pool of plant- and microbe-accessible N arises from conceptual gaps in current explanations about the fundamental mechanisms that drive N bioavailability; these stem in large part from failing to accurately account for the organic component of the soil N cycle and its biogeochemical drivers.

The need to emphasize organic N is reminiscent of the impetus that led to developments in how the soil organic carbon (SOC) cycle is conceptualized. In the twentieth century, researchers theorized that the inherent chemical recalcitrance of carbon (C) to decomposition controlled SOC turnover, but evidence from the last two or more decades reveals that microbes can degrade even the most complex molecules (Gleixner et al. 2001, 2002; Rasse et al. 2006) and that, in the context of overall soil organic matter (SOM) dynamics, recalcitrance only temporarily controls microbial SOC processing rates. Instead, SOC persistence largely emerges from constraints that the soil mineral matrix imposes on microbial access to substrates (Kleber et al. 2011; Schimel and Schaeffer 2012) and SOC dynamics are better predicted by biological and physical controls on C transfer between different SOC pools (Six et al. 2006; Grandy and Neff 2008), motivating several recent soil C cycling models to explicitly incorporate soil physical fractions (Sulman et al. 2014; Wieder et al. 2015; Abramoff et al. 2018; Kyker-Snowman et al. 2019). The fate of ON similarly relies on how associations with minerals regulate access to N-containing molecules (Lavallee et al. 2020) which are in turn regulated by biologically mediated chemical and physical processes that have yet to be integrated into the soil N paradigm (Darrouzet-Nardi and Weintraub 2014).

Here, we aim to unify advances in the understanding of N transformations by developing a new, testable conceptual model of organic bioavailable N in soil. We ground our model in two commonly measured SOM pools: particulate organic matter (POM) and mineral-associated organic matter (MAOM), capturing the importance of both the depolymerization of N-containing molecules (Schimel and Bennett 2004) and mineral sorption-desorption (Sollins et al. 1996; Jilling et al. 2018). We highlight how microbial physiological traits shape the fate of N once it is taken up by microbes. Finally, consistent with Drinkwater and Snapp’s (2007) agroecosystem N model and insights into priming mechanisms (e.g. Cheng and Coleman 1990; Dijkstra and Cheng 2007; Phillips et al. 2012; Zhu et al. 2014), we explicitly address the role of plants and their interactions with minerals and microbes in mobilizing N. Below we outline our new model, synthesize relevant new data, and examine some implications of our model in fertilized agroecosystems, aggrading and degrading soils, and under a changing global climate.

## Bioavailable nitrogen: conceptual model

**Fig. 1:**
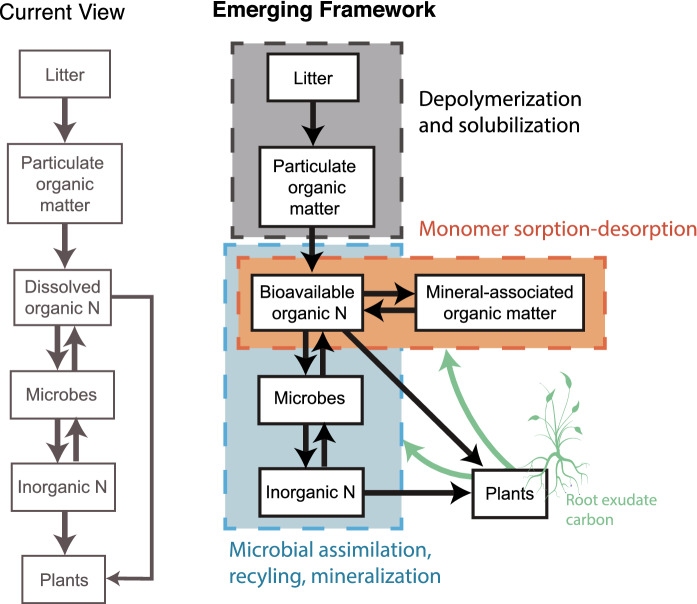
Conceptual models illustrating current and emerging frameworks of soil bioavailable N cycling. The emerging model emphasizes three major compartments: (1) depolymerization and solubilization, in grey; (2) interactions between bioavailable organic N and minerals, in orange, and (3) microbial assimilation, recycling, and mineralization of organic N, in blue. Black arrows represent the direction of N flow between pools. Green arrows indicate the direction of plant root exudate C flow. This model does not attempt to capture all steps in the process (see *Future Directions*). The "current view" is adapted from Schimel and Bennett 2004. (Color figure online)

As with previous conceptual frameworks, our model (Fig. 1) traces the flow of N from SOM through bioavailable ON (via depolymerization; Schimel and Bennett 2004) to microbial biomass and finally into inorganic N via mineralization. While depolymerization can limit the overall rate of SON cycling, here we focus on its role in supplying N directly to MAOM and indirectly to MAOM through microbes. Importantly, our model separately considers POM and MAOM; this establishes sorption and desorption as an important sink and source of bioavailable N. MAOM forms through associations with the mineral matrix where mineral properties: (i) determine the chemistry and stability of these organo-mineral interactions (e.g. Parfitt et al. 1997; Baldock and Skjemstad 2000; Krull et al. 2004; Grandy et al. 2009; Abelenda et al. 2011; Buurman and Roscoe 2011); (ii) dictate each soil’s potential to accumulate SOM; and (iii) regulate the sorption/desorption dynamics that govern the supply of bioavailable N from MAOM. Nitrogen from microbial biomass can recycle back into bioavailable N and SOM, providing a mechanism for soil N retention and reuse. We thus detail how the physiology of soil microbial communities shapes the amount and partitioning of N flow between bioavailable N, microbial biomass, MAOM, and inorganic N through uptake, assimilation, recycling, and mineralization.

**Fig. 2:**
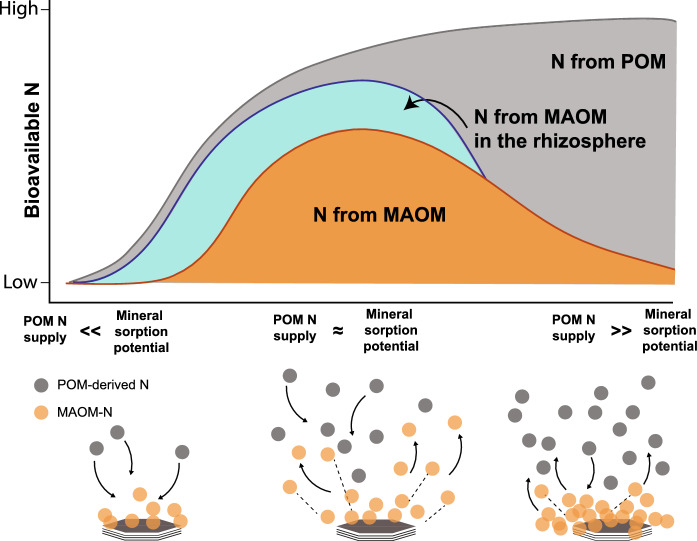
Conceptual illustration of how soil bioavailable N and its source (POM vs. MAOM) depend on the ratio of the incoming supply of POM-N to mineral sorption potential, defined as net sorption (i.e. greater gross sorption than gross desorption) of organic N. Stacked curves depict the amount of bioavailable N derived from POM sources (gray), MAOM sources in bulk soil (orange), and MAOM sources under the influence of plant-microbe interactions in the rhizosphere (turquoise). Low POM N supply relative to mineral sorption potential (POM N supply << Mineral sorption potential) will favor sorption and result in low N bioavailability. Bioavailable N from MAOM peaks in soils where POM N supply and mineral sorption potential are in relative balance and overall N bioavailability is moderate-to-high (POM N supply ≈ Mineral sorption potential). High relative POM N supply makes POM the principal source of bioavailable N and results in high N bioavailability (POM N supply >> Mineral sorption potential). The specific dynamics of bioavailable N will vary depending on the physical and chemical properties of POM and MAOM, total SOM content, soil mineralogy, and the specific nature of microbial communities and plant-soil interactions. (Color figure online)

The proportion of bioavailable N derived from POM or MAOM (Fig. 2) depends on the ratio between N mobilized from POM, via depolymerization and solubilization, versus the potential for mineral sorption. The latter arises from the properties of soil colloids, soil texture, and the overall chemistry and quantity of MAOM and N in the soil solution (Rillig et al. 2007; Dippold et al. 2014). This framework emphasizes the role of minerals in intercepting, immobilizing, and releasing bioavailable N via sorption and desorption processes. When mineral sorption potential is high relative to the rate of POM deposition, much of the mobile N entering MAOM associates strongly with minerals and thus is less able to desorb and become available to plants and microbes. In these conditions, the mineral sorptive potential principally establishes the equilibrium of sorbed vs. dissolved N. As the POM-N supply increases relative to mineral sorption potential, the MAOM pool’s likelihood of exchange with the bulk solution increases. At this stage, MAOM will also include more organic molecules that associate loosely with mineral surfaces or other MAOM and are therefore more accessible to microbes (and, in N-limited systems, plants; e.g. Kleber et al. 2007; Jilling et al. 2018). When high levels of N supply from POM greatly exceed mineral sorption potential, high concentrations of bioavailable N from POM result. Some bioavailable MAOM-N exists as a result of exchange with POM-N in solution, but the high concentration of POM-derived bioavailable N should slow MAOM desorption.

It is worth noting that these bioavailable N dynamics will be modified by stoichiometry-driven processes like co-metabolism and priming in which, for example, microbes might degrade POM for its C but MAOM for its N, or might liberate N from MAOM as a side effect of mining for phosphorus or micronutrients (Blagodatsky et al. 2010; Di Lonardo et al. 2017; Čapek et al. 2018). Our model also incorporates the understanding that plants are not passive players gathering up the leftovers of microbial mineralization; rather, through direct actions and by triggering microbes to act, plants can shape N cycling (see discussion in *Model details: Monomer sorption-desorption*). Thus, superimposed on the source-sink dynamics of the POM-N supply and mineral sorption potential, the plant-microbe system can increase MAOM-N provisioning in the rhizosphere (Fig. 2, *N from MAOM in the rhizosphere*).

How differences in POM and MAOM alter bioavailable N can be hypothetically illustrated by comparing the contrasting properties of Mollisols and Aridisols. Tallgrass prairie Mollisols of the Central US are characterized by high mineral sorption capacity and a high rate of N supply from POM (Liu et al. 2012; Fig. 2: *POM N supply ≈ Mineral sorption potential*). In these soils, our framework suggests that incoming POM-N will become MAOM, but as bioavailable N supply increases that organic N will form loose, easily exchangeable associations with other MAOM; while a small portion of POM-derived N will remain in solution, most bioavailable N will still come from the more labile fraction of the large MAOM pool. In soils with very high POM inputs or very low sorptive capacity, POM will directly supply the majority of bioavailable N (Fig. 2: *POM N supply* >> *Mineral sorption potential*). On the other end of the spectrum (Fig. 2: *POM N supply* << *Mineral sorption potential*), some Aridisols supply little N from POM; this leaves unfulfilled mineral sorptive capacity and results in meager amounts of bioavailable N. An individual soil’s mineral sorption potential can also shift over time as MAOM pools accrete or degrade as a function of variation in POM-N inputs and removal of bioavailable N from the system by plants, microbes, and environmental losses. We further discuss MAOM and POM dynamics during soil degradation and regeneration in the Applications section of this article.

## Model details

### Depolymerization and solubilization

N from POM first enters the bioavailable pool when its N-containing polymers break down into soluble organic N oligomers and monomers, including amino acids (AAs, Fig. 1, grey box). Traditionally, the primary control on N supply to plants was thought to be the derivation of ammonium (NH_4_^+^) from ON, i.e. N mineralization, a stance dating to as far back as the late 1800s (Russell and Russell 1950; Harmsen and Van Schreven 1955). In 2004, Schimel and Bennet articulated an emerging consensus that considered depolymerization, rather than inorganic N production, as the rate-limiting step for N bioavailability (see also Ladd and Paul 1973). In fact 50–75% of dissolved ON in soil solution is composed of small, bioavailable peptides and amino acids (Yu et al. 2002). With a half-life of only minutes to hours, free amino acids form a small but very dynamic pool of ON in soils and plant litter that are quickly taken up by plants and microbes or sorbed to minerals (Kielland et al. 2007; Jones et al. 2009; Wanek et al. 2010; Mooshammer et al. 2012). Microbes consumed free amino acids at a rate > 8 times greater than ammonium and nitrate during leaf litter decomposition, as measured by a ^15^N-AA pool dilution technique for quantifying gross rates of protein depolymerization and amino acid uptake (Wanek et al. 2010). Other ON monomers, oligomers, and small peptides have similarly rapid turnover (Hill et al. 2012; Farrell et al. 2013; Hu et al. 2018; Warren 2019; Ma et al. 2020).

New lines of research are exploring the major controls over protein depolymerization and amino acid cycling. Substrate availability limits protein depolymerization in subsoil and plant litter (Mooshammer et al. 2012; Ma et al. 2020), and explained 60–70% of variation in gross protein depolymerization across several land uses (Noll et al. 2019b). Depolymerization is one strategy by which microbes may acquire N in response to N limitation or C excess: nutrient scarcity induces microbes to preferentially decompose N-bearing polymers from leaf litter (Reuter et al. 2020), and labile C additions increased gross depolymerization rates (Noll et al. 2019b). Alternately, depolymerization could be a C acquisition strategy: in some studies, excess C lowered amino acid and peptide uptake (Farrell et al. 2014; Yang et al. 2020) and increasing litter C:N was associated with lower rates of gross depolymerization (Mooshammer et al. 2012).

Other evidence contradicts the hypothesis that substrate or nutrient scarcity should increase depolymerization. Substrate concentration was not found to influence breakdown of amino sugar polymers (Hu et al. 2018) or other N-containing polymers in topsoil or incubated forest soil (Wild et al. 2019; Ma et al. 2020). Many studies have found no effect of organic or inorganic N additions on gross soil peptide or amino acid cycling (Farrell et al. 2014; Wild et al. 2019; Noll et al. 2019a; Yang et al. 2020). Inconsistent observations about how stoichiometry relates to depolymerization could be due to the fact that depolymerization products supply microbes with both C and N, or due to system level microbial community adaptations that alleviate nutrient constraints (Kaiser et al. 2014).

The identity of the decomposers may also influence protein depolymerization. Saprotrophic fungi may degrade N polymers faster than bacteria (Hobbie and Hobbie 2012) and mycorrhizal fungi can influence decomposition dynamics (Frey 2019). Microbial communities may differ in extracellular protease expression (Puissant et al. 2019), amino acid scavenging (Moe 2013), and cellular peptide transport (Li et al. 2020a). Tight microbial recycling of microbial necromass N could also maintain depolymerization rates regardless of fluctuations in inputs of new substrate (Cissé et al. 2020). Further, the turnover of ON may depend on which forms and chemical structures of ON are available for microbial decomposition (Geisseler and Horwath 2014) and the extent to which interactions with minerals protect substrates from enzymatic attack (Rillig et al. 2007; Wang et al. 2020a). Soil mineral composition has been found to influence gross depolymerization and amino acid cycling rates (Noll et al. 2019b; Hu et al. 2020), and soil physiochemical properties that influence substrate entrapment in small pores and aggregate structures will also regulate the breakdown of N polymers into bioavailable N (Six et al. 2000; Grandy and Robertson 2007; Smith et al. 2017).

### Sorption-desorption of bioavailable organic N

The majority of total soil N resides in mineral-associated organic matter fractions (Fig. 1, orange box), which are defined based on particle size (< 53 um) and/or density (< 1.7 g cm^3^). MAOM was long considered inaccessible to microbes and plants because radiocarbon data indicate it has very slow average turnover times (centuries to millennia; Fabrizzi et al. 2003; Denef et al. 2013; Paul 2016); therefore, MAOM has been broadly characterized as a sink, and POM as source, of bioavailable N. However, POM fractions store only a small proportion (< 20%) of total ON in mineral soils and POM can even act as a sink for N in early stages of decomposition due to its relatively high C:N ratio (Whalen et al. 2000; Fornara et al. 2011; St. Luce et al. 2011). In contrast, MAOM is enriched in microbial products (Schmidt et al. 2011; Miltner et al. 2012; Kopittke et al. 2018) and low-molecular-weight plant compounds (Haddix et al. 2016) and thus possesses a low C:N ratio (Sollins et al. 2006), which generally promotes N mineralization via microbial N mining (Sollins et al. 1984; Whalen et al. 2000; Jilling et al. 2018).

Incubations of SOM fractions show higher rates of N mineralization from MAOM than POM (Bimüller et al. 2014), supporting recent evidence that MAOM is heterogeneous in chemistry and function, and some MAOM is relatively accessible (Mikutta and Kaiser 2011; Torn et al. 2013). Mineral-associated fractions can exhibit short-term (< 5 years) changes in C and N content (Heckman et al. 2013; von Haden et al. 2019; Jilling et al. 2020), indicating a fraction of this pool cycles on relatively rapid time scales. Soil capacity to accumulate MAOM has also been linked to aboveground productivity: Cates and Ruark (2017) observed a positive association between the non-aggregated silt and clay fraction and crop yield. Similarly, both POM and MAOM have been positively associated with select measures of N availability and crop performance (Wade et al. 2018; Jilling et al. 2020). Because MAOM includes both easily exchangeable and highly persistent fractions, minerals can retain organic compounds—building SOM—while also supplying bioavailable N.

MAOM formation from POM can be fast: minerals quickly stabilize POM-derived N, as demonstrated by the rapid transfer of ^15^ N-labelled residues into MAOM fractions (Kölbl et al. 2006; Bosshard et al. 2008; Poirier et al. 2020; T.M. Bowles, unpublished data). N associated with minerals can be remobilized, in part because MAOM often accrues not as a continuous layer but rather as patches that vertically extend outward from mineral surfaces (Vogel et al. 2014) or bind only via weak bonds and may thus be more likely to exchange or interact with the soil solution (Kleber et al. 2007; Gao et al. 2020). Desorption potential of ON also differs between clay mineral types due to their variation in surface area and charge characteristics. Yet microbes are able to access some ON associated with minerals—even from iron and aluminum oxides that bind ON more strongly than most phyllosilicate clays (Kaiser and Zech 2000; Kleber et al. 2005; Mikutta et al. 2005).

In recent years, evidence has emerged that rhizosphere processes mobilize MAOM-N (Jilling et al. 2018; Fig. 2: *N from MAOM in the rhizosphere*). Root C inputs and the release of H^+^ and OH^−^ during cation or anion uptake cause dramatic, localized shifts in both pH and soil solution chemistry that alter organic matter sorption onto, or mobilization from, mineral surfaces (Avena and Koopal 1998; Rashad et al. 2010; Kleber et al. 2015; Singh et al. 2016). Strong ligands such as oxalate and citrate released by roots can mobilize MAOM-N by exchanging for organic compounds held in metal–organic complexes (Kleber et al. 2015) or by dissolving minerals such as iron and aluminum (hydr)oxides (Xyla et al. 1992; Vempati et al. 1995). Plant roots can secrete enzymes including extracellular proteases to break large N polymers into bioavailable N (Tornkvist et al. 2019). Plant rhizodeposits include large amounts of photosynthetically fixed carbon (e.g. Litton et al. 2007) and simple, low molecular weight exudates (Dennis et al. 2010) that influence mineral solubility (Hinsinger and Courchesne 2007; Calvaruso et al. 2014; Keiluweit et al. 2015).

In addition to these direct effects, rhizodeposition can indirectly undermine the stability of mineral-SOM associations (Keiluweit et al. 2015; Jilling et al. 2018). Root inputs can “prime” MAOM- N mobilization indirectly by stimulating microbial activity, which generates acidity and depletes oxygen. This can alter the redox state of metals, causing MAOM-N to be released (Fischer et al. 1989; Grybos et al. 2009; Husson 2013; Buettner et al. 2014). These root deposits can also stimulate microbes to produce extracellular enzymes, notably oxidases that are effective at destabilizing SOM (Sinsabaugh 2009; Phillips et al. 2011; Zhu et al. 2014; Partavian et al. 2015; Kieloaho et al. 2016; Wang et al. 2020b).

### Microbial organic N turnover: uptake, assimilation, recycling, mineralization

The physiological traits of microbes shape how N flows through the microbial compartment (Fig. 1, blue box) by affecting extracellular depolymerization, cellular uptake, metabolic and biosynthetic allocation, and finally plant uptake or environmental losses in inorganic and organic forms. First, microbes acquire ON at rates that depend on the characteristics of (a) the extracellular enzymes that produce small peptides and N monomers from larger substrates, and (b) the membrane transport proteins that move the resulting bioavailable ON into microbial cells. These two classes of proteins can vary between microbes in functionally relevant characteristics including abundance, specificity, efficiency, inducibility, and the energetic costs required for microbes to build and operate them. If microbes take up peptides rather than monomers they can invest less in ON decomposition (Hobbie and Hobbie 2012) though this could also require more specialized and expensive transporters (Davis et al. 2005). Microbes can assimilate ON more efficiently if they have traits that confer stoichiometric or metabolic flexibility, for example by responding to molecule or element limitation by switching to alternative energy or biosynthesis pathways that use more favorable substrate molecules (Smith and Chapman 2010). Adaptive traits like luxury N consumption and storage can accrue N in cellular biomass (Frost et al. 2005), while competitive or cooperative traits can release N into the soil environment in compounds like antibiotics and the protein components of extracellular polymeric substances (Allison 2005; Ren et al. 2015; Estrela et al. 2019; Cai et al. 2019; Garcia-Garcera and Rocha 2020). Microbes can lose N passively as concentration gradients drive reverse diffusion through permease sites (Krämer 1994; Button 1998) to an extent that likely varies between microbes with different N uptake systems. Physiological traits that confer stress resistance may limit microbial ON loss by reducing membrane disruptions.

SON recycling and MAOM-N accumulation will arise in part from the outcome of microbial N-allocation to biomass, excreted biomolecular products, and mineralized N. Initial evidence suggests that greater recycling of microbial N within the soil environment lessens inorganic N waste excretion by microbes (Zhang et al. 2019). Fast microbial growth provides more opportunities for recycling of microbial N within the soil as microbial lysates, necromass, and biomolecular products are re-incorporated into microbial biomass or sorbed to soil minerals. External factors that accelerate microbial biomass turnover and release microbial N into the soil environment include seasonal changes in temperature and moisture; predation by micro- and mesofauna and viruses; and the chemistry, amount, and variability of plant root inputs (Clarholm 1985; Singh et al. 1989; Lipson et al. 1999; Scheu 2002; Kuzyakov and Mason-Jones 2018; Emerson 2019). Greater microbial carbon use efficiency (CUE) accelerates the accumulation of N-rich microbial products and necromass in soil (Kallenbach et al. 2016, 2019) by increasing the amount of microbial biomass produced per unit of substrate (Manzoni et al. 2012; Geyer et al. 2019) and may itself be driven by microbial community composition (Kallenbach et al. 2019; Domeignoz-Horta et al. 2020). The soil environment can modify microbial N allocation. For example, the proportion of assimilated ON that microbes released as waste NH_4_^+^ decreased in suboxic conditions but increased with temperature (Zhang et al. 2019), and was moderately greater under long-term warming and drought (Wild et al. 2018). Further elucidating how microbial physiology responds to environmental controls will be critical in predicting when N will be mineralized versus recycled within SON pools.

Microbial release of N waste also depends on the elemental imbalance between microbial biomass and substrate resources (Sterner and Elser 2002; Li et al. 2020b). Soil microbial biomass has a relatively fixed average biomass C:N ratio of 8:1 (Cleveland and Liptzin 2007; Kallenbach and Grandy 2011); stoichiometric theory predicts microbes achieve this by offloading excess substrate C or N as CO_2_ or NH_4_^+^ waste (Mooshammer et al. 2014b). Meanwhile, microbial substrates in soil environments range from very N-rich (C:N ratio of e.g. < 5:1) to N-poor containing little N (e.g. > 100:1) or no N (e.g. cellulose; Sinsabaugh et al. 2016), leading to a wide range in the intensity of the stoichiometric imbalance between microbes and SOM resources. Across soil and litter samples, Mooshammer et al. (2014a) noted much greater release of inorganic N waste when resource C:N was similar to microbial C:N, which decreased as the gap between resource and microbial C:N widened, approaching minimum release of inorganic N at a microbe-resource stoichiometric imbalance of about four-fold. These observations suggest that a higher proportion of N will be mineralized from MAOM than from POM substrates due to MAOM’s lower average C:N ratio.

## Applications and future directions

Our model can be applied to consider the delivery of bioavailable N from POM and MAOM in fertilized agroecosystems and disturbed systems (main text) as well as across seasons and in response to changes in soil moisture (Online Appendix).

### POM and MAOM in degrading and aggrading soils

In the terms of our conceptual model, a degrading soil is one in which MAOM-N desorption rates exceed MAOM-N sorption rates; consequently, mineral sorption potential increases, and the soil shifts left along the x-axis in Fig. 2. MAOM depletion could occur due to increased desorption rates, for example from N-mining by plants, microbes, and plant–microbe consortia, or due to decreased sorption rates due to decreasing POM inputs. Indeed, in many degrading soils including those undergoing desertification or conversion to intensive agriculture, POM reserves are expected to decline, leaving MAOM as the primary source of bioavailable N without resupply, further depleting MAOM-N in an accelerating process of soil degradation. As disturbance continues to empty the MAOM pool and the mineral sink strengthens, we expect MAOM-N to become increasingly inaccessible. Soils with low sorption potential often rely primarily on POM to supply bioavailable N and are vulnerable to degradation due to the speed at which POM decomposes, particularly when new POM inputs also decline.

Refilling POM pools by restoring productive aboveground plant communities—for example through reforestation, perennialization, or cover cropping—can regenerate the ability of soils with low sorption potential to supply bioavailable N. Over time, large and consistent POM inputs can also replenish the degraded MAOM pools of soils with high mineral sorption potential. However, building MAOM pools requires a large amount of N per C because of its low C:N ratio (Cotrufo et al. 2019), perhaps in part because the nitrogenous moieties in ON are particularly reactive with mineral sorption sites (Omoike and Chorover 2006; Lambert 2008) and seem to play an important role in forming organo-mineral complexes (Kleber et al. 2007). Therefore, MAOM may accrue more quickly from materials that are highly processed by microbes, from low C:N materials, and from higher C:N materials that are deposited simultaneously with a source of inorganic and/or organic N. For example, recent studies observed that manure had a greater capacity to build MAOM than crop residue (Samson et al. 2020), and that inorganic N, manure, and soybean additions each increased microbial conversion of maize residues to MAOM (Gillespie et al. 2014). Inputs that improve plant and microbial uptake of ON monomers (Ma et al. 2018) could increase recycling and retention of ON within soil, and particularly MAOM, pools. The ability to regenerate MAOM will also depend on whether soil conditions support the conversion of plant litter or exogenous organic inputs to MAOM, for example whether new ON inputs are in physical contact with minerals or accessible to microbial enzymes.

### Accounting for MAOM-N in agroecosystem nutrient management

In agroecosystems, global fertilizer nitrogen use efficiency (NUE) remains stubbornly low at around 40%, and must nearly double by 2050 to meet predicted food and environmental demands (Zhang et al. 2015). The modest success of technological solutions focused on fertilizer management (Xia et al. 2017; Norton and Ouyang 2019) reveals the shortcomings of a narrow focus on managing inorganic N. Our model adds to calls for active management of SON (Gardner and Drinkwater 2009; Lin et al. 2016; Yan et al. 2020) and suggests that future agronomic research should seek to develop ways to enhance N supply from POM and MAOM when plant demand is high, but equally, to rebuild those SON pools during non-growing or fallow seasons.

Sites will require management practices tailored to their specific mineralogical properties and POM and MAOM concentrations. Sites with high mineral sorption capacity but low POM (Fig. 2, *POM N supply* << *Mineral sorption potential*) will supply little bioavailable N to crops, but have great potential to provide MAOM-N if management can increase POM inputs and their conversion to MAOM. Sites where POM-N supply and mineral sorption potential are balanced will need practices aimed toward maintenance of the POM and MAOM pools. Soils with very low mineral sorption potential or very high POM-N supply are prone to sizeable N losses (Fig. 2, *POM N supply* >> *Mineral sorption potential*), and will benefit most from strategies that can absorb excess bioavailable N by increasing soil charge potential or metal cation concentrations to enhance MAOM-N storage, and by enlarging microbial biomass pools.

**Fig. 3:**
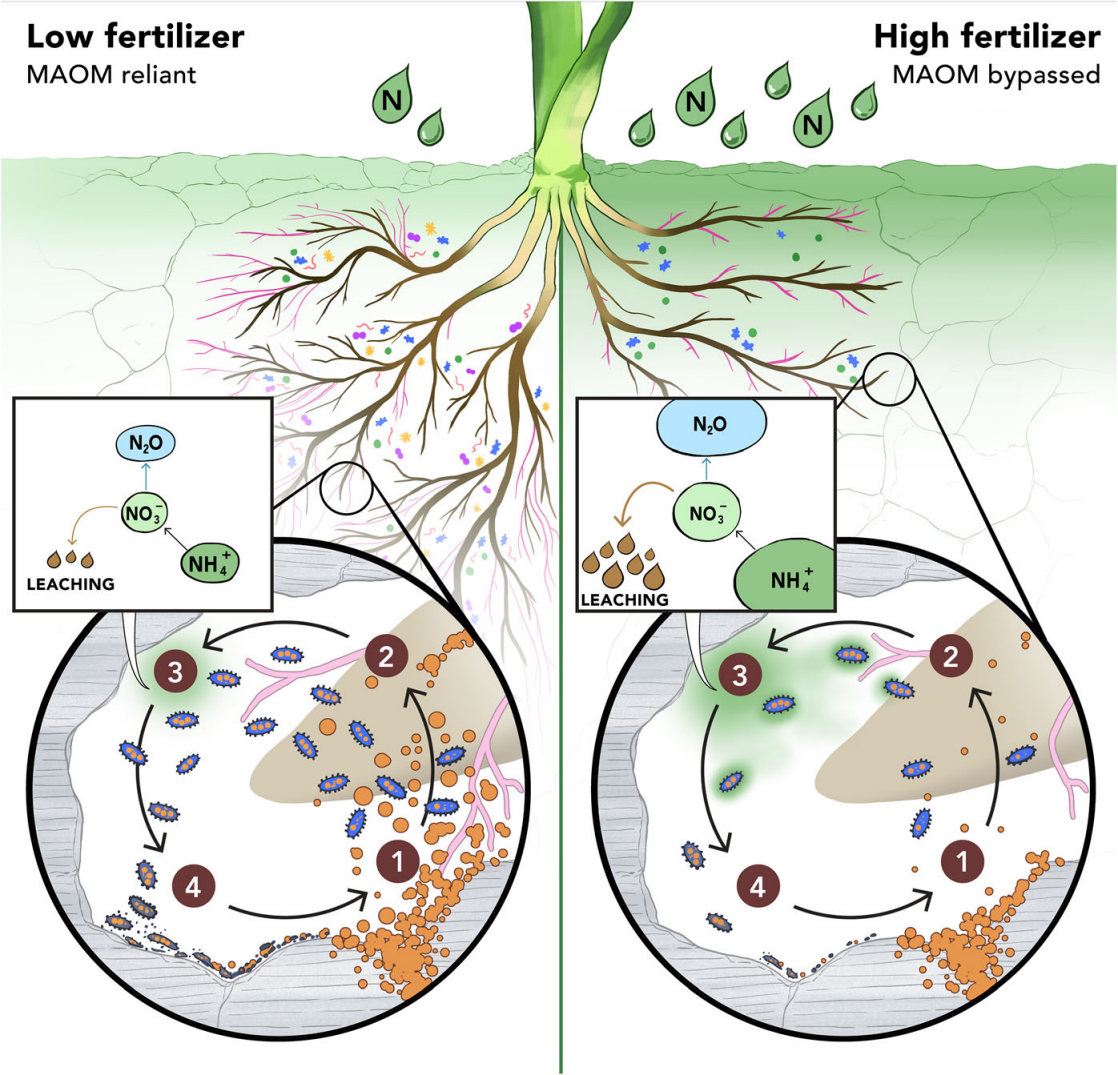
Potential fertilizer impacts on bioavailable N supply from MAOM in soils with adequate MAOM-N (i.e. Figure 2, POM N supply ≈ Mineral sorption potential). Left: Modest, economical fertilizer application (lighter green gradient) incentivizes plants to invest in root production and associations with mycorrhizae (pink). Resulting plant-microbe-mineral interactions in minimally fertilized soils (1) liberate more bioavailable N from MAOM (orange); (2) increase microbial biomass; (3) produce less microbial ammonium waste and contribute less to N losses; and (4) increase necromass inputs that can replenish MAOM-N pools. Right: Heavy fertilizer application (darker green gradient) disrupts these plant-microbe-mineral interactions. (Color figure online)

We expect inorganic N applications will substantially alter MAOM-N mobilization (Fig. 3, right) by suppressing the biological mechanisms that mobilize MAOM. Inorganic N can decrease plant-microbe mobilization of MAOM in the rhizosphere by selecting for microbes that are poorer decomposers or that are less responsive to root inputs; by shifting microbial communities to have fewer fungi; or by lowering overall microbial biomass (Treseder 2008; Fierer et al. 2012; Morrison et al. 2018; Jia et al. 2020). N fertilizer reduces mycorrhizal fungi that extend root surface area (Phillips et al. 2012; Morrison et al. 2016); N fertilizer can also accelerate the activity of hydrolytic enzymes such as beta-1,4-glucosidase, while reducing the activity of oxidative enzymes that mobilize MAOM (Grandy et al. 2008; Jilling et al. 2018; Chen et al. 2020). The acidifying effect of nitrification has also been theorized to reduce MAOM pools (Averill and Waring 2018). Thus, our framework is in line with Drinkwater and Snapp’s (2007) argument that it is critical to recouple C and N cycles in agroecosystems to maximize yields while minimizing economic and environmental costs of N excess. For example, it suggests that MAOM pools can be best enriched by organic fertilizers like animal manure, crop residues, or compost (Leinweber and Reuter 1992; Chen et al. 2019; Huang et al. 2019; Xu et al. 2020), and that green manures and cover crops can convert inorganic N into POM inputs that both supply bioavailable N and build MAOM.

In addition to ongoing agronomic research that seeks to minimize inorganic N inputs, our model encourages development of strategies to engage the plant-microbe-soil interactions that accelerate N provisioning when and where N demand is high, such as during rapid vegetative growth phases and in the rhizosphere. For instance, crop breeding can select for plants with greater or better timed exudation of organic acids and root-secreted proteases; increased ability to interact with soil microbes like mycorrhizal fungi and to induce rhizosphere microbes to mine N from MAOM; more active amino acid importer proteins and a greater capacity to alter root growth phenotypes in response to changes in soil amino acid concentrations; and increased plant use of soil peptides (Forsum et al. 2008; Moe 2013; Moreau et al. 2019; Tornkvist et al. 2019; Preece and Peñuelas 2020). Agroecologists can also seek to develop management regimes that select for soil microbial communities that respond more to plant inputs, and are less influenced by soil inorganic N concentrations.

For managed ecosystems, we suggest seeking strategies that prioritize building MAOM pools and that re-conceptualize POM pools as a more secondary concern whose main import is to feed the microbes that generate MAOM. Soil management regimes should also select for microbes that can efficiently convert POM-N—and even excess organic and inorganic fertilizer N—into microbial products that build MAOM-N. At the same time, these ideal soil microbes should readily depolymerize ON substrates and mobilize ON from minerals to generate bioavailable N. We posit that these microbial communities should be highly active to further increase the turnover and exchange of MAOM-N. A better understanding of soil microbial physiology related to ON cycling can ensure that the balance between these microbial effects will supply N but not deplete MAOM (Janzen 2006). Such developments in agronomic tools will lead to more tightly coupled plant-soil N cycling in which bioavailable N supply better coincides with plant N demand (Bowles et al. 2015).

### Future directions

Our conceptual model of bioavailable N suggests that we need to address important knowledge gaps and increase research effort in several areas. Very little is known about the controls on gross protein depolymerization, and even less is known about how microbial taxa differ in their contributions to these controls. Insights in this area will also improve our understanding of bioavailable N dynamics in organic soils, such as histosols, which fall outside the scope of our model. Upstream of depolymerization, soil biota including soil meso- and microfauna physically fragment litter into POM and deposit N-rich feces (Wickings and Grandy 2011). How these animals influence MAOM formation and turnover remains to be determined (David 2014). Leachate from fresh litter is a direct and potentially large source of bioavailable N (Rinkes et al. 2014), and it may differ in its chemistry from compounds originating in POM or microbial products in ways that influence its associations with minerals. Insoluble macromolecules of plant and microbial origin also associate with minerals (Lehmann and Kleber 2015) and, because they are subject to both desorption and depolymerization, likely have multiple controls. Aggregation and other types of physical occlusion (e.g. low pore connectivity or soil moisture) may further modify the dynamics of MAOM turnover and ON bioavailability. Finally, plants are both sinks for bioavailable N and sources of ON in the form of litter deposits, and differences in plant-microbe-soil interactions could cause plants to vary in how they influence bioavailable N cycling across environments, especially in ecosystems where plants also assimilate especially large amounts of organic N such as the Arctic (Sorensen et al. 2008).

Our model recognizes the importance of microbial physiology in partitioning N between SOM and inorganic pools. There is much to learn about how the flow of ON through the microbial pool is shaped by microbial identity and genomic potential, community structure and interactions, and constraints of the soil environment. Do different microbes or consortia vary in their expression of ON degrading and uptake transport proteins, in their growth rates and efficiencies, or in their metabolic flexibility and how they allocate N from organic sources? How does recycling of bioavailable N between MAOM and microbes alter its chemistry and future bioavailability? How do microbes alter their use of ON in response to stress, particularly the types of stress they will increasingly face in a changing climate? Use of appropriate measures of microbial growth efficiency (Frey et al. 2013; Geyer et al. 2016) and the increasing power of functional omics and meta-omics technologies (Sergaki et al. 2018; Pinu et al. 2019; Nannipieri et al. 2020; Ichihashi et al. 2020; Tang and Aristilde 2020; Naylor et al. 2020) are advancing this exciting new theme in soil biogeochemical research.

Given that soil mineral composition likely drives at least part of the site specificity so often found in studies of SON, we need to clarify the ways in which minerals affect bioavailable N cycling. There remain uncertainties around the most basic interactions between various bioavailable N species and different minerals, the strength of these interactions, and their vulnerability to disruption (Schulten and Schnitzer 1997; Kleber et al. 2015). We will require more detailed characterization of the ways different ON polymers and monomers interact chemically with one another, with inorganic N and other solutes including metallic ions, and with enzymes and redox processes. Researchers have gained new insights into the 3-D architecture of organo-mineral interactions (Mueller et al. 2013) and how organic compounds fractionate between soil mineral pools (Heckman et al. 2013); they have learned that some minerals preferentially sorb dissolved ON over compounds lacking N. How these insights relate to bioavailable N deserves more detailed inquiry. At the same time, we recognize that “MAOM” originated as an operational term for organic matter attached to dense and/or small (typically < 53 µm) particles (Cambardella and Elliott 1992; Jastrow 1996), but that this fraction can incidentally include very small POM fragments and insoluble ON (Lavallee et al. 2020). The emerging conceptual understanding of MAOM as a pool of potentially soluble ON of diverse chemical makeup calls for more sophisticated characterization of this soil fraction.

## Conclusion

We present a new framework of bioavailable N cycling based on the interactions between organic N depolymerization, mineral sorption-desorption dynamics, and the actions of plants and microbes. New research, enabled by methodological advances of the last decade, has revealed depolymerization to be a dynamic process that drives substantial fluxes of bioavailable N from POM; this organic N subsequently associates with soil minerals to form MAOM, a large and heterogeneous pool of SOM enriched in nutrients that roots and microbes can actively mine. Our framework suggests that the flow of bioavailable N from MAOM is based on the relative balance between POM-N inputs and the soil’s mineral sorption potential, further shaped by plant-microbe interactions and environmental conditions. Microbial physiological traits substantially impact the entire bioavailable N cycle. By accounting for MAOM-N dynamics, we can develop agricultural management strategies that better minimize N pollution while reaching crop yield goals. As the SON paradigm is reshaped—the way SOC paradigm has been reshaped over the last two decades—new avenues will open to understanding the cycling of bioavailable N.

## Supplementary Information

Below is the link to the electronic supplementary material.

Supplementary file1 (DOCX 16 kb)

Supplementary file2 (EPS 1685 kb)

## Abbreviations

AA: Amino acid
C: Carbon
CUE: Carbon use efficiency
MAOM: Mineral associated organic matter
N: Nitrogen
NUE: Nitrogen use efficiency
ON: Organic nitrogen
POM: Particulate organic matter
SOC: Soil organic carbon
SOM: Soil organic matter
SON: Soil organic nitrogen
