# Supplementary material for: A holistic framework integrating plant-microbe-mineral regulation of soil bioavailable nitrogen
Source: Biogeochemistry. 2021 May 6;154(2):211–29. doi: 10.1007/s10533-021-00793-9 (PMC8570341; doi:10.1007/s10533-021-00793-9)
Supplement: Supplementary file 1 — Supplementary file1 (DOCX 16 kb) [file 10533_2021_793_MOESM1_ESM.docx]

### Appendix to Daly *et al.*

#### Nitrogen sources under variable water regimes

Climate models predict future moisture regimes that are more variable and extreme for many regions, which will greatly impact N availability and losses in natural and managed ecosystems (Sinha et al. 2017; Bowles et al. 2018). Compared to POM, we expect that MAOM will be a more consistent and less loss-prone source of bioavailable N across soil moisture conditions (**Figure 4a**) so that soil ecosystems with greater mineral sorption capacity may be better able to maintain plant productivity under water stress. POM decomposes best in moist soils: at intermediate soil water content, the microbes and enzymes that surround organic particles can access enough moisture to optimally depolymerize and degrade organic matter. Dry soils strongly limit microbe and enzyme activity and nutrient diffusion in pores, while in saturated soils slow oxygen diffusion limits microbial activity and POM decomposition.

**Fig. 4** *Potential soil moisture (a) and seasonal (b) effects on the supply of bioavailable N from MAOM (orange) and POM (gray). (a) Bioavailable N originates from MAOM under water-limiting conditions and is relatively high and even across the remainder of the soil moisture range (solid orange line). N does not begin to become bioavailable from POM until soil water content is low-to-moderate, after which bioavailable N from POM peaks at moderate soil water content and declines with water saturation (solid gray line). A larger proportion of bioavailable N from POM has the potential to contribute to environmental losses (dashed gray line) while a lower proportion of bioavailable N from MAOM is predicted to contribute to environmental losses (dashed orange line). (b) N bioavailability from POM (gray curve) differs from N bioavailability from MAOM (orange curve) in response to seasonal temperature changes, as further described in the text. Bioavailability of N from MAOM is driven more by the plant-microbe-mineral network, while bioavailability of N from POM is driven more by decomposition dynamics that arise from interactions between inputs, temperature, and moisture.*

MAOM-N should desorb over a much wider range of soil moisture. Minerals retain a thin water film in all but the driest conditions but POM desiccates more quickly; therefore, in very dry but not completely dry soils, we might expect that the POM-decomposing activities of soil biota slow more drastically than sorption-desorption processes in the narrow zone of moisture remaining around minerals. Because roots and mycorrhizal hyphae drive the release of N from MAOM in the rhizosphere, bioavailable N from MAOM is immediately available at the root or hypha surface. Many microbes live on or near mineral surfaces, and even free-moving microbes become concentrated in water films on minerals as soils dry, allowing them to access water and MAOM-N in soils with very little overall moisture. Likewise, proteolytic and oxidative enzymes accumulate on and around mineral surfaces and become concentrated in the water films of drying soils. In low-moisture conditions, plants may rapidly deplete MAOM-N mobilized in the rhizosphere, but as re-wetting restores mass flow, any bioavailable N released from MAOM in bulk soil can diffuse toward N sinks including roots and microbial hot spots.

Environmental losses of MAOM-N and POM-N across gradients of soil water availability are also expected to differ. In dry soils, MAOM-N loss potential is likely nil: leaching cannot occur, microbial activity is low, and high oxygen availability discourages gaseous N production. As soils are first re-wet, any MAOM-N not captured by plants or incorporated into microbial biomass will be vulnerable to losses; but across the remainder of the soil moisture spectrum, from moderate moisture through water saturation, loss of MAOM-N should remain low and at a constant proportion of MAOM-N production. This is due to our hypothesis that N is released from MAOM partly in response to targeted actions by plants and microbes that are poised to immediately use it. POM-derived N supply can exceed MAOM under optimal moisture, though environmental losses are also likely higher over intermediate and high moisture contents because of spatial and temporal disconnects between bioavailable N production and uptake.

Such relationships between water availability and the fate of different sources of bioavailable N are accentuated by plant N uptake capacity. Plant N uptake is optimal at field capacity, and inhibited with severe drying, but otherwise relatively constant across the soil moisture gradient (Wuest and Cassman 2004, Hegde and Srinivas 1990 Irrig Sci), similar to our expectations for MAOM-N mobilization. This makes MAOM-N a good source of bioavailable N while minimizing losses. POM-N decomposition, on the other hand, is optimal only over a narrow range of soil moisture, making its dynamics less parallel to those of plants and thus more prone to exceed or fall short of plant uptake capacity, potentially leading to N pollution. Finally, we expect MAOM has a greater water holding capacity than POM, and is therefore better able to buffer soil moisture to make severe drying—and therefore rewetting—events less frequent.

#### Nitrogen sources across seasons

As soils thaw in spring, microbial and plant activity ramps up; we predict that this results in a burst of N released from both POM and MAOM (**Figure 4b**). Bioavailable N is expected to be released from MAOM at a relatively steady rate throughout the growing season in response to activation by plants and microbes, which could be strongest during periods of greatest plant N need (e.g. mid-to-late summer). POM is likely depleted of bioavailable N early in the season and can only contribute modestly to soil dissolved ON throughout the summer with fluctuations driven mostly by moisture and temperature (e.g. mild increase with late summer heat). By fall, we expect plant-driven MAOM-N release to slow as plants senesce/die, but we also expect new N to be released with decomposition of POM from fresh litter inputs, which could prime concomitant MAOM-N release. Indeed, throughout the growing season, POM-N and MAOM-N could to some extent prime one another’s release.
